# A Case of Abdominal Hysterectomy in Which the Ureter Was Resected and Implanted into the Bladder

**Published:** 1894-09

**Authors:** Charles B. Penrose

**Affiliations:** Philadelphia, Pa.; Professor of Gynecology in the University of Pennsylvania, Philadelphia


### A CASE OF ABDOMINAL HYSTERECTOMY IN WHICH THE URETER WAS RESECTED AND IMPLANTED INTO THE BLADDER.

BY CHARLES B. PENROSE, M.D.,

Professor of Gynecology in the University of Pennsylvania, Philadelphia.

I report this case because the immediate implantation into the bladder of a ureter, which has been divided during a celiotomy, is a rare proceeding.

The patient was a white woman, forty years old, who had a scirrhous cancer of the cervix uteri. The growth extended as high as the internal os, and infiltrated the left broad ligament, in a dense hard mass, to a distance of about one inch from the cervix. There was no involvement of the vagina. There were no symptoms of obstruction of the ureter.

Celiotomy was performed at the Gynecean Hospital in July, 1893. It was found that the left ureter passed directly through the hard mass in the left broad ligament; and in order to remove completely all diseased tissue, it was necessary to excise about one inch of the ureter—the portion involved in the broad ligament.

After the ureter had been cut away at the vaginal junction, the distal end of the ureter was ligated with silk; the vagina was closed; the peritoneum was sutured over the seat of operation as much as possible; and the proximal portion of the ureter was then implanted into the body of the bladder. The operation was similar to, and was derived from, that used by Dr. Van Hook for uniting a ureter after complete transverse division, by lateral implantation of the proximal into the distal portion (*Journ. Amer. Med. Assoc.*, March 4, 1893). An incision was made antero-posteriorly in the body of the bladder somewhat less than one-half inch in length. A needle armed with fine silk was passed through the bladder-wall from without in, at a point about one-third inch from the edge of the incision on the right, and brought out through the incision. It was then passed through the right wall of the ureter close to the extremity, carried back through the incision in the bladder and passed through the bladder-wall from within out, close to its point of entrance. A similar suture was passed on the left side of the incision in the bladder and through the left side of the wall of the divided ureter. Traction on these sutures dragged the ureter into the bladder, and when tied, they held it in this position.

The loose peritoneum, which formed a partial investment to the ureter, was drawn down and sutured to the peritoneum of the bladder by a continuous silk suture around the line of union of ureter with bladder. The abdomen was closed without drain. A soft rubber catheter was introduced through the urethra and was retained for three days. The patient made an unusually easy recovery.

There were no symptoms of bladder or renal disturbance. The quantity of urine passed was as follows: 10 ounces in the first twenty-four hours; 26 ounces in the second twenty-four hours; 22 ounces in the third twenty-four hours; and 38 ounces in the fourth twenty-four hours.

She left the hospital twenty days after operation. Her physician wrote me in December that she was perfectly well and doing her own housework. He wrote again in February—over six months after the operation—that she was suffering with pain in the right iliac region—perhaps a recurrence of the disease.

She has at no time presented any symptom whatever of disease of the urinary organs.

Our text-books on surgery advise that, in case the ureter be torn or cut across during the removal of an abdominal tumor, the renal end must be brought out through an opening made for this purpose in the loin. And in some instances nephrectomy has been performed for the relief of such an accident.

The clinical and experimental researches of Dr. Van Hook, of Chicago (paper read at the Forty-fourth Annual Meeting of the American Medical Association, June, 1893); a recently-reported case of Dr. H. A. Kelly, when a divided ureter was immediately united by lateral implantation of the proximal into the distal portion, and the case just reported, go to show that the advice of our surgical text-books should be modified; and that, if the patient is able to endure a slightly prolonged operation, and the anatomical conditions are suitable, it is better immediately to implant the proximal portion of the ureter into the distal portion, or into the bladder.

